# Editorial: Functional foods: adding value to food

**DOI:** 10.3389/fnut.2024.1466003

**Published:** 2024-09-09

**Authors:** Esra Capanoglu, Simin Feng, Srinivas Janaswamy

**Affiliations:** ^1^Food Engineering Department, Faculty of Chemical & Metallurgical Engineering, Istanbul Technical University, Istanbul, Türkiye; ^2^Department of Food Science and Technology, Zhejiang University of Technology, Hangzhou, Zhejiang, China; ^3^Department of Dairy and Food Science, South Dakota State University, Brookings, SD, United States

**Keywords:** functional food, bioactive compounds, bioavailability, health, dietary supplement, food processing

Functional foods are designed to provide health benefits in addition to supplying essential nutrients. They effectively prevent various diseases due to their vitamin, mineral, bioactive, probiotic, and fiber content, and potentially improve the quality of life. The concept has attracted attention due to the relationship between human nutrition and health. These foods are specially formulated and produced to present enhanced efficiency. Lifestyle-related health problems such as cardiovascular diseases, diabetes, obesity, and gastrointestinal disorders, increase the demand for healthy, nutritious, and safe food products. Indeed, functional foods have the potential to improve people's eating and drinking habits. These traits, coupled with the positive results obtained, have increased consumer awareness, and hence, the functional foods sector has proliferated. The global functional food market is deemed to reach hundreds of billions of dollars in the coming years with a significant growth curve. According to the 2018 data, its market was ~170 billion US dollars, while this value has approximately doubled in 5 years and is estimated to reach 300 billion USD in 2025. The market is primarily driven by North America, Europe, Asia, and the Pacific (1–4).

This Research Topic covers cutting-edge research on (i) the design and development of food products with novel and improved functional properties, (ii) identifying the challenges that may be encountered during the integration of bioactive components into food products, (iii) identifying technologies that can compensate with the challenges, and (iv) investigating the functional activities of new food ingredients. Emphasizing the potential benefits and health effects of the developed functional food ingredients and determining the market value of functional food products, challenges associated with the processing of functional foods and alternative strategies are also covered. These clear and realistic objectives shed light on the design and development of functional food products using bioactive components with high nutritional value and advanced bioavailability properties. This Research Topic presents the latest research and review articles on the consumption of bioactive compounds, related health effects, and their correlation with demographic properties.

Kwon from Mokwon University, Republic of Korea, studied the multifaceted analysis using data from the nationally representative health and nutrition review survey conducted in South Korea. Within the scope of the study, the relationship between the consumption of dietary supplements and society's socioeconomic factors was determined. The individuals with high socioeconomic status in the context of education, health, and income were indicated to be more likely to use dietary supplements consistently. It further emphasized the need to support equitable access to dietary supplements for different socioeconomic groups. The team of Zheng et al. from Wenzhou Academy of Agricultural Sciences, China, investigated the effects of black tea extract on D-galactose-induced skin quality and signs of aging in mice. The results indicated that black tea extract significantly increases skin elasticity, hydration, and antioxidant enzyme activity by reducing oxidative stress. However, future studies are needed to understand the mechanisms of the relevant bioactive components and determine the potential of the integration of these ingredients into cosmetic products. Liao et al. from Southeast University, China, investigated the potential hypoglycemic effects of pea protein hydrolysate in mice with dietary and chemically induced diabetes. The results indicated that pea protein hydrolysate lowered blood sugar and could provide improvements in insulin sensitivity levels suggesting that pea protein hydrolysate could be an effective nutritional ingredient in treating diabetes. In addition, the potential effect of functional foods on blood glucose regulation was exemplified, and the necessity of clinical trials was emphasized. Nicolescu et al. at the University of Medicine and Pharmacy, Romania, compiled the health effects and bioaccessibility of various phytochemicals used in nutraceuticals and functional foods. The study focused on how food interactions affect bioaccessibility in the context of matrix and processing methods. Their results showed that bioaccessibility values varied significantly between different phytochemicals. The significance of using different food processing techniques to enhance the efficiency of phytochemicals and process optimization was also emphasized.

Even though the significance and health benefits have been well established, there are also several concerns regarding functional foods. There is a need for further research on the cumulative risks and safety of the bioactives in these products. In addition, the interactions of different functional compounds in the context of the food matrix need to be studied in more detail. More importantly, indiscriminately defining the developed products as functional is a controversial issue. Thus, determining whether such products have a biological effect needs to be investigated and we believe this will become one of the essential fields of study in the near future. Harmonizing and reconsidering legal regulations is another critical issue that needs attention. Studies on these areas will directly contribute to the design, processing, and marketing of functional foods, and the future holds significant prospects.
